# Glucose

**Published:** 1881-08

**Authors:** 


### GLUCOSE.

PROFESSOR H. W. Wiley, in the *Popular Science Monthly*, records the amazing progress of the glucose industry. There are twenty immense glucose factories either built or in progress of construction. Already a capital of over $2,000,000 is invested in the business. The daily consumption of corn for the manufacture of glucose is about 35,000 bushels, and the annual amount about 11,000,000. All the factories have sprung up in the last twelve years. They are run day and night, Sundays included, to meet the demand, and still fail to supply it. Notwithstanding these notorious facts it is almost impossible to find anybody willing to admit that he buys glucose or uses it for any purpose. Its existence is never made known by newspaper advertisements or placards. It is never bottled, boxed or barreled under its own name. It is as carefully screened from public view as nitro-glycerine. There is no doubt that glucose is largely used in the preparation of table syrups and candies, for brewing and for artificial honey. Nine-tenths of the cheap candies are made of glucose. It is hard to find a table syrup wholly free from this adulteration. Glucose is said to be present in most of the beer now brewed. The bee himself is not as busy as the glucose manipulator. The latter puts up a "honey" of which the waxen cells are made of paraffine and the contents consist of snow-white glucose. The fraudulent comb and honey are handsomer than the finest white clover "Vermont," and can be sold at half the price at a greater profit. This story would be deemed incredible but for the positive assurance of men like Professor Wiley. To the question, "Is glucose wholesome?" the professor replies that it all depends on the presence or absence of sulphuric acid, lime and copper, which are apt to find their way into the article through imperfect chemical treatment.

